# A134 TRANSITION FROM PEDIATRIC TO ADULT CARE WITH EOSINOPHILIC ESOPHAGITIS: A PATIENT EXPERIENCE SURVEY

**DOI:** 10.1093/jcag/gwae059.134

**Published:** 2025-02-10

**Authors:** T T Hoang, V Avinashi, H Ko

**Affiliations:** The University of British Columbia Faculty of Medicine, Vancouver, BC, Canada; The University of British Columbia Faculty of Medicine, Vancouver, BC, Canada; The University of British Columbia Faculty of Medicine, Vancouver, BC, Canada

## Abstract

**Background:**

Healthcare transition for pediatric patients with chronic gastrointestinal diseases is a coordinated process that requires multidisciplinary collaboration to improve an otherwise challenging time for patients and their families. While this process has been explored in other areas such as inflammatory bowel disease, the transition experience has not been well characterized in eosinophilic esophagitis (EoE).

**Aims:**

To better understand and characterize the experiences and attitudes of patients transition from pediatric to adult care with EoE in British Columbia.

**Methods:**

We conducted an online, anonymous, self-administered, 10–15-minute survey of adult patients who had recently transitioned to adult care. EoE patients age ≥ 19 who were discharged from the EoE clinic at BC Children’s Hospital within the past three years and referred to adult gastroenterology at the Digestive Disease’s Centre in Vancouver, British Columbia were invited to participate. The survey included demographics and questions assessing patients’ pre-transition readiness based on a modified Transition Readiness Assessment Questionnaire (TRAQ), and post-transition challenges via a 5-point Likert scale. Free text entries were included to allow for qualitative thematic analysis of patients’ responses.

**Results:**

Of the twenty patients identified who met inclusion criteria, eleven (55%) completed the survey. Only four (36.3%) patients at the time of transition felt prepared to graduate into adult care. While six (54.5%) reported feeling satisfied with the transition process, four (36.3%) felt that the process was more challenging than expected. Seven (63.6%) patients felt that communication between their pediatric and adult physicians was inadequate. Moreover, four (36.3%) patients felt they lost access to non-gastroenterology specialists such as allergists and pediatricians, as well as allied health care members after transition into adult care. Thematic analysis of patients’ free-text responses revealed that disjointed communication between healthcare teams, new therapeutic relationships, and loss of access to medical experts were three concerning themes for transitioning patients. Ten (90.9%) patients agreed a joint clinic between pediatric and adult gastroenterology would have improved their transition experience.

**Conclusions:**

These preliminary data from our ongoing study highlight the challenges associated with EoE pediatric-to-adult transition. Given the identified unifying themes of inadequate communication between care providers, a joint transition EoE clinic between pediatric and adult physicians would be a potential solution to significantly streamline this process.

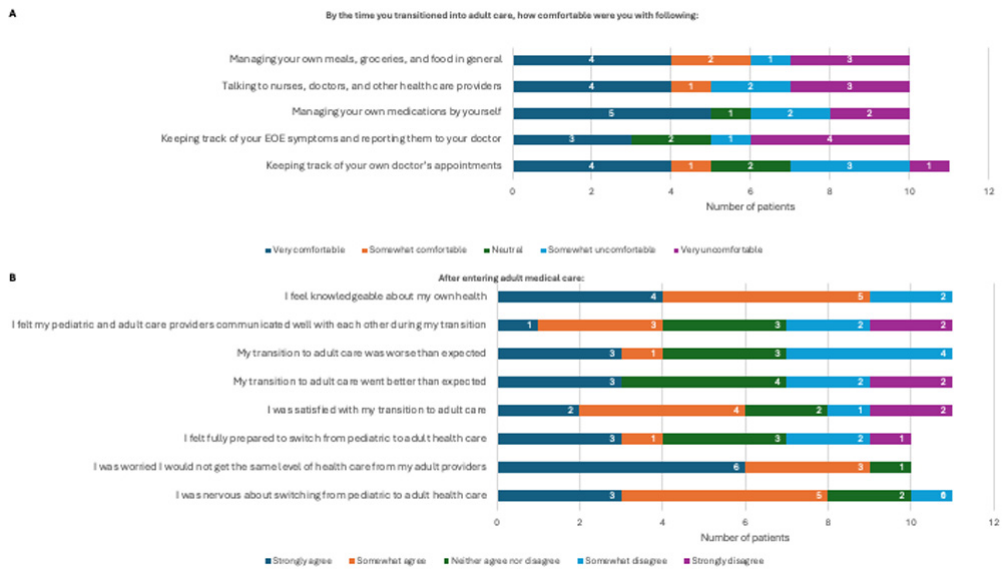

Figure 1: Patient attitudes regarding (A) pretransition readiness using a modified Transition Readiness Assessment Questionnaire (TRAQ) and (B) post-transition experience.

**Funding Agencies:**

None

